# Multiple General Anesthesia in Children: A Systematic Review of Its Effect on Neurodevelopment

**DOI:** 10.3390/jpm13050867

**Published:** 2023-05-21

**Authors:** Giacomo Colletti, Mattia Di Bartolomeo, Sara Negrello, Roy G. Geronemus, Bernard Cohen, Luigi Chiarini, Alexandre Anesi, Raimondo Feminò, Ilaria Mariotti, Gregory M. Levitin, Linda Rozell-Shannon, Riccardo Nocini

**Affiliations:** 1 Department of Medical and Surgical Sciences for Children & Adults, Cranio-Maxillo-Facial Surgery, University of Modena and Reggio Emilia, Largo del Pozzo 71, 41124 Modena, Italy; giacomo.colletti@gmail.com (G.C.); luigi.chiarini@unimore.it (L.C.); alexandre.anesi@unimore.it (A.A.); 2 The Vascular Birthmark Foundation, P.O. Box 106, Latham, NY 12110, USA; drlevitin@gmail.com (G.M.L.); vbfpresident@gmail.com (L.R.-S.); 3 Unit of Dentistry and Maxillo-Facial Surgery, Surgery, Dentistry, Maternity and Infant Department, University of Verona, P.le L.A. Scuro 10, 37134 Verona, Italy; 4 Cranio-Maxillo-Facial Surgery Unit, University Hospital of Modena, 41124 Modena, Italy; negrellosara86@gmail.com; 5 Laser and Skin Surgery Center of New York, New York, NY 10016, USA; rgeronemus@laserskinsurgery.com; 6 Pediatric Dermatology and Cutaneous Laser Center, Johns Hopkins University, Baltimore, MD 21287, USA; bcohena@jhmi.edu; 7 Anesthesia and Intensive Care Unit, Department of General and Specialist Surgeries, University Hospital of Modena, 41124 Modena, Italy; raimondofemino@gmail.com; 8 Onco-Hematology Paediatric Unit, Department of Medical and Surgical Sciences for Mothers, Children and Adults, University Hospital of Modena, 41124 Modena, Italy; mariotti.ilaria@aou.mo.it; 9 Section of Ear Nose and Throat (ENT), Department of Surgical Sciences, Dentistry, Gynecology and Pediatrics, University of Verona, 37124 Verona, Italy; riccardo.nocini@gmail.com

**Keywords:** multiple general anesthesia, neurodevelopment, neurocognition, early surgery

## Abstract

The effect of multiple general anesthesia (mGA) procedures administered in early life is a critical theme and has led the Food and Drug Administration (FDA) to issue an alert. This systematic review seeks to explore the potential effects on neurodevelopment of mGA on patients under 4 years. The Medline, Embase and Web of Science databases were searched for publications up to 31 March 2021. The databases were searched for publications regarding “children multiple general anesthesia OR pediatric multiple general anesthesia”. Case reports, animal studies and expert opinions were excluded. Systematic reviews were not included, but they were screened to identify any possible additional information. A total of 3156 studies were identified. After removing the duplicates, screening the remaining records and analyzing the systematic reviews’ bibliography, 10 studies were considered suitable for inclusion. Comprehensively, a total cohort of 264.759 unexposed children and 11.027 exposed children were assessed for neurodevelopmental outcomes. Only one paper did not find any statistically significant difference between exposed and unexposed children in terms of neurodevelopmental alterations. Controlled studies on mGA administered before 4 years of age support that there might be a greater risk of neurodevelopmental delay in children receiving mGA, warranting the need for careful risk/benefit considerations.

## 1. Introduction

The issue of the risk related to the use of general anesthesia (GA) in the pediatric population is of recent and relevant interest. As pointed out by Shi et al., approximately 15% of children in the USA undergo GA before 3 years of age, and approximately 4% receive multiple general anesthesia (mGA) or a single general anesthesia (sGA) that lasts more than 3 h before the age of 3 [1]. In December of 2016, the Food and Drug Administration (FDA) issued an alert and then, in August 2018, issued an update on the potential risks related to GA during the most formative period for brain development [2,3]. The background for this alert is a large database of preclinical and clinical studies, which have highlighted the possible role of early GA in determining a delay in neurocognitive development [4,5,6,7,8,9,10,11,12,13,14]. Therefore, the term “anesthesia-related neurotoxicity” was coined, but its definition is not yet well established, and its consequences include a wide set of manifestations:

- Learning disorders, which include language, cognitive and motor deficits;
- Behavioral disorders, which comprise autism spectrum disorder (ASD) and attention-deficit hyperactivity disorders (ADHDs);
- Diminished results in academic performances.

As of today, a clear picture of the consequences of early exposure to anesthetics has not yet been clarified, even in preclinical models. The multiple facets of neurodevelopment are reflected in the heterogeneity of the outcome measures that are used in clinical practice [15]. In fact, there is no tool that can globally describe all of the issues considered. It must be said that a developmental delay is not intended as a decrease in overall intelligence but, for instance, it can also manifest only as a behavioral disorder or a decrease in motor abilities. Some of the most frequently used instruments include the following:

- Full-Scale Intelligence Quotient (FSIQ) score of the Wechsler Abbreviated Scale of Intelligence [16];
- Early Development Instrument (EDI), composed of 104 items to assess physical health and well-being, language and cognitive skills, social competence, emotional development, and communication ability and general knowledge [17];
- International Classification of Diseases, Clinical Modification–Coded Diagnoses (ICD-CM), which include language, cognitive and behavioral disorders [18,19];
- Child Behavior Check List (CBCL), a tool to identify behavioral problems in children, which is part of the Achenbach System of Empirically Based Assessment [20];
- Clinical Evaluation of Language Fundamentals (CELF), which presents subdomains divided into receptive language (CELF-R), expressive language, speaking skills (CELF-E) and an overall score (CELF-T) [21];
- Colorado Learning Difficulties Questionnaire (CLDQ) [22];
- Raven’s Colored Progressive Matrices (CPM), which is one of the oldest tests, originally published in 1938;
- The Ages and Stages Questionnaire (ASQ), an inventory that has to be completed by parents;
- Academic performances, which can take into consideration school grades or intelligence quotient (IQ) scores;
- Tests for motor abilities, such as the McCarron Assessment of Neuromuscular Development (MAND), the heel-to-toe walking (which measures dynamic balance) or the peg-placing tasks (to test manual dexterity).

Over the years, significant improvements in terms of monitoring, medications and anesthesiological techniques have been obtained in the field of pediatric anesthesia, thus leading to improvements in recovery after surgical interventions and overall survival following early surgeries [23]. A crucial point has been the recent conceptualization of pediatric anesthesia, which should be approached differently compared to the one for adults, especially from a pharmacological point of view [24]. Nonetheless, despite the controversy surrounding the FDA alert, there is still no international consensus on the effects of early exposure to anesthetic drugs. Several narrative reviews have tried to paint a picture of the situation [25,26,27]. An interesting analysis was performed by Wang et al. in 2014. This included a systematic review and meta-regression on the effect of GA on neurodevelopment in children. The authors showed that exposure to GA before age 4 is associated with a hazard ratio (HR) of 1.25 for developing an adverse neurodevelopmental outcome, which grows to 1.75 for those who have been exposed multiple times to GA before the age of 4. Both of these results were statistically significant, with a *p*-value < 0.0001 [28]. Ing et al., on the other hand, performed a systematic review and meta-analysis of prospective studies that evaluated the effects of an sGA, highlighting that there is no difference in general intelligence between exposed and unexposed patients [29]. In a recent study performed by Song et al., an evaluation of the risk of developing ADHD in children exposed to general anesthesia with endotracheal intubation was performed. The results obtained suggested that those children exposed to multiple general anesthesia procedures or for a longer duration were more susceptible to developing ADHD. It has to be said that the median age of the cohort was 3.8; thus, some treated patients were older than four at the time of treatment [30]. Recently, Grabowski et al. tried to answer, in a systematic review, some key questions concerning the correlation between early exposure to GA and neurocognitive effects, such as its dose dependency, the existence of a critical window of danger, if specific agents expose to a higher risk and the presence of alternatives to GA [31]. 

Given the inconsistent findings on this subject, this systematic review focuses on the risk related to the exposure to multiple GAs in the early neurodevelopmental period. A thorough examination of the literature on the subject was performed, and the results were summarized and compared in order to check the current evidence and discuss the implications for clinical practice.

## 2. Materials and Methods

This systematic review was performed by searching the Medline, Embase and Web of Science databases. The search string used, based on Boolean operators, was “children multiple general anesthesia OR pediatric multiple general anesthesia”, including all publications up to and including 31 March 2021. The study was conducted following the Preferred Reporting Items for Systematic Criteria Reviews and Meta-Analyses (PRISMA) [32,33]. The review protocol was not registered in any online database.

The main research question is whether the exposure to mGA at an early age could cause long-term developmental delay compared to unexposed children. The population considered in the systematic review was that of children undergoing mGA before the age of 4. An initial screening of articles was conducted independently by two authors, eliminating the papers not strictly relevant to the research question. Case reports, animal studies and expert opinions were excluded. Systematic reviews were not included as selected studies, but they were, nonetheless, screened in order to find any additional record not identified by the main search.

Subsequently, the following inclusion criteria were applied:

- Retrospective, prospective or bidirectional case-control studies in children exposed to at least two GAs before age 4;
- Studies presenting neurodevelopmental outcome measurement;
- English-language research papers.

The exclusion criteria, on the other hand, were:

- Age at exposure to GA greater than 4 years of age;
- Studies that exclusively investigated patients exposed to a single GA;
- Uncontrolled studies;
- Subanalyses of other studies.

After this process, the remaining papers have been catalogued, extracting the following data: exposed and unexposed children’s cohorts, age at exposure, outcome, risk measurement and its possible significance. 

## 3. Results

A total of 3156 records were identified by searching the Embase, Medline and Web of Science databases. After removing the duplicates, the remaining 2252 titles were screened to remove all studies that did not match the focus of this review. The references of the systematic reviews identified in the first search were analyzed, finding 13 more papers to be assessed. The full text of the resulting 38 papers were then examined, and 10 studies could, finally, be included in the qualitative analysis [34,35,36,37,38,39,40,41,42,43]. Additionally, 28 papers were excluded, according to the aforementioned criteria. Some papers used a dataset that was then included in a wider study. For example, in 2014, Ko et al. studied children registered in the National Health Insurance Research Database (NHIRD) of Taiwan, who were born between 1 January 2001 and 31 December 2005 [44]. In 2020, Feng et al. evaluated all children in the NHIRD who had a medical event from 1 January 2000 to 31 December 2013 [43]. Moreover, some studies were subjected to subanalyses (e.g., the Mayo Anesthesia Safety in Kids (MASK) study) [40,45,46].

**Figure 1:**
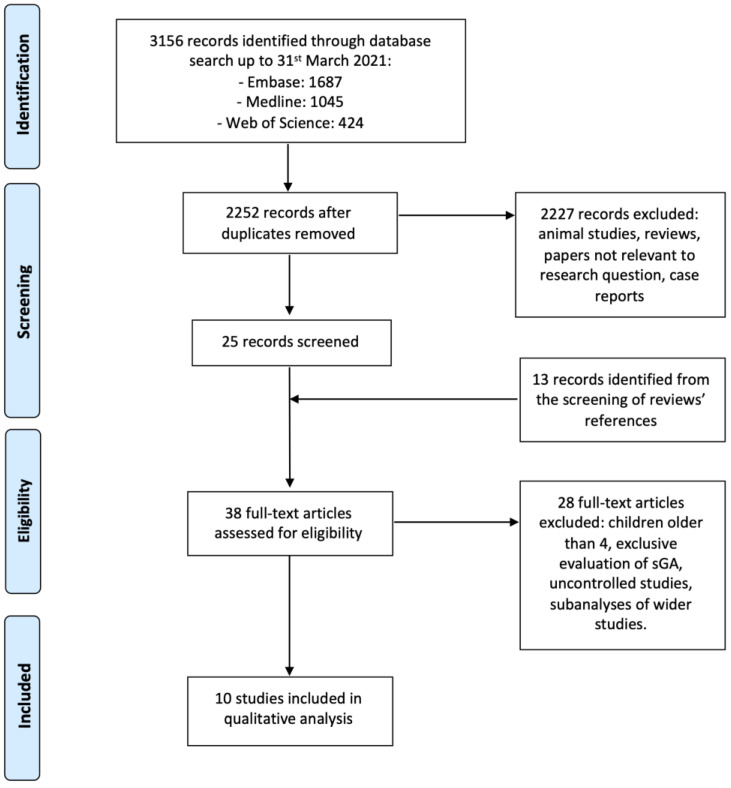
Search strategy according to PRISMA guidelines.

The full search strategy is summarized in Figure 1.

Among the 10 selected studies, there were a total of 264.759 unexposed children and 11.027 exposed children. The oldest paper was published in 2009, while the most recent was published in 2021.

The results are summarized in Table 1.

Four studies distinguished the results for children who underwent two GAs and children who underwent three or more GAs. Two studies enrolled only children younger than 2 years old, four studies enrolled children under age 3, and four studies enrolled children under age 4.

The outcome measures were very heterogenous. Two papers studied ADHD prevalence in exposed and unexposed cohorts, while two papers evaluated the diagnosis of developmental and/or behavioral disorders according to the ICD-9-CM codes. One paper analyzed academic performances (school grades at age 16) as the main endpoint together with IQ scores at the age of 18. The remaining papers evaluated neurodevelopmental disorders by means of a wide battery of neuropsychological tests, such as the FSIQ, EDI, CELF, Child Communication Checklist, CBCL and others.

Only one paper did not find any statistically significant difference between exposed and unexposed children. Of note, this held true for those children who underwent only two GAs before the age of 4. In the same cohort, a significative difference in terms of academic performances was noted between controls and children who underwent three or more GAs. As clearly evidenced in Table 1, all of the other papers found a statistically significant difference in terms of neurodevelopmental delay between children who were exposed to mGA and the controls. These differences might be true for all or some of the outcomes taken into consideration.

The main risk of bias was related to the underlying pathologies of exposed children. In fact, the reason behind the administration of several GAs might be a confounding factor in the analysis of the result. Nevertheless, it has to be said that all of the included papers only enrolled children without a previous intellectual disability, neurological disorders or any other clear risk factor for neurodevelopmental delay. Another bias is linked to the lack of a unique tool to measure the risk of anesthesia-related neurotoxicity. As of today, a homogeneity in evaluating neurodevelopmental delay is not possible and further studies are needed to create a dedicated evaluation tool. 

## 4. Discussion

As evidenced in this systematic review, exposure to several GAs in the early phase of neurodevelopment may pose a risk to the child for subsequent neurocognitive impairments. Given the sensitivity of the issue and the frailty of the patients treated, this theme has to be analyzed from several perspectives.

First, one of the questions of greatest interest is inherent to the pediatric age at risk, namely, the definition of a “window of danger” or “window of vulnerability”. This term refers to the age group in which exposure to mGA can lead to long-term neurocognitive impairments. Generally speaking, it is currently believed that the most sensitive period is before the age of 3 or 4 [2,3]. At this stage of development, in fact, the brain is subjected to important remodeling, and anesthetic drugs could interfere with the processes of synaptogenesis, neurogenesis, and survival of neuronal cells. An interesting finding by Graham et al. was that a stratification by age did not show a difference in neurodevelopmental delay in children exposed before age 2 compared to those exposed between ages 2 and 4 [38]. Nevertheless, the potential deleterious effects of anesthesia should also be investigated during other periods of brain maturation (such as the first trimester or puberty). For example, the risks of prenatal anesthetic exposure are topics of main concern trending in current research, both in preclinical and clinical settings [47,48,49]. Therefore, continuous monitoring is essential, and the study of animal models can help in understanding where to focus efforts. At the same time, preclinical studies do not guarantee the same validity as studies on humans in this field, because animal neurobiology is very different from that of humans [50]. This implies caution in accepting results obtained from preclinical models, although the latter represents the basis of the approach to neurobiology [51]. 

It appears evident from this systematic review that multiple exposures to GA can determine an increased risk of neurotoxicity related to anesthesia. However, a quantification of this risk is not easy to define. The first difficulty, in this sense, is linked to properly defining the problem. For neurodevelopment, in fact, we have seen how many spheres are involved [15], and there is a lack of a standard instrument to assess them all. In this systematic review, it was decided to include studies that used different tests to measure neurocognitive delays. Despite each test having its own peculiarities, the results appear to point to the same direction. For example, although the predisposing factors still remain unknown, a secondary analysis performed in the context of the MASK study has shown an association between multiple general anesthesia procedures and a deficit in some neuropsychological skills. In particular, a significant decrease in motor, processing speed and visual–motor integration skills has been observed [46].

Furthermore, subjective factors also come into play in this area, especially those linked to the parental perception of a possible deficit. As shown in the MASK study, parental reported outcomes may play a role and be significant in assessing the complexity of pediatric neurodevelopment [40]. The involvement of parents or guardians in the decision-making process is a key point in the FDA’s warning [3]. 

Another important factor to consider is the underlying pathologies that may play a role as confounding factors. All selected papers enrolled children who did not have clear risk factors for neurodevelopmental delay. At the same time, it cannot be excluded that the comorbidities affecting these young patients might play an active role in determining a neurocognitive disorder [3]. As noted by Lo and Kalish, inflammation during the surgical intervention also might affect brain development, which could be negatively influenced by the cumulative action of systemic cytokines. At the same time, they also claim that uncontrolled post-operative pain might have detrimental effects on neurodevelopment, as the persistent activation of nociceptors can be dangerous [52]. Therefore, inadequate post-operative analgesia and anti-inflammatory management can also be counted as confounding factors. Moreover, social confounding factors might take part in the process. Adverse social conditions, familiar environments, parents’ level of educational or household income might have an impact on neurodevelopmental delay. However, these variables were taken into consideration when assessing case-control homogeneity in the selected papers.

Other factors to be considered are the anesthetic techniques and various drugs used. They differ from center to center and from patient to patient. Therefore, it makes it more difficult to obtain a clear homogeneity. In this systematic review, a time frame for article inclusion was not predetermined, but the oldest included study was published in 2008, therefore, reflecting the current clinical management of anesthetic procedures. Regarding the drugs used and their possible adverse consequences, the FDA, in its warning, required some anesthetic and sedation drugs to be labeled with information regarding their potential detrimental effects on neurodevelopment [3].

Given the risks highlighted by this systematic review, some final considerations could be made.

The ultimate goal must be the child’s short-term and long-term well-being, and, as pointed out by the FDA, cooperation between parents and caregivers must be the basis of every decision [2,3]. Among all of the questions, this confrontation must also address those regarding the timing of treatments and the need for them. Given the risks highlighted, however, it would perhaps be more prudent to perform the procedures under local or regional anesthesia, when possible and safe (e.g., laser treatments for small vascular malformations). Providing the possibility of quick and painless treatments can be a way to prevent such consequences. Moreover, along with anesthesia-related neurotoxicity, other factors may affect pediatric neurodevelopmental delay. These factors might be both clinical (such as intraoperative hypotension or hyperoxia) or psychological (such as long-term hospitalization or separation from the family). Obviously, as already stated, there is still the need for prospective studies centered on the topic, with a comprehensive approach that can provide clear answers to the issues previously discussed. As also pointed out by Ing and Bellinger, prospective studies can help to select the specific outcomes to be studied and to focus on appropriate instruments to measure them. Nonetheless, a critical problem is represented by the difficulty of enrolling a large and controlled cohort study that should also be followed up and tested for several years [53]. At the same time, a careful subgroup analysis should be performed to select those confounding factors that might also affect neurodevelopmental outcomes. A recent review by Keunen et al. concluded that the key research topics should be the patterns of neonatal white matter injuries and the correlations among direct anesthetic neurotoxicity, inflammation and alteration of brain perfusion determined by immature vessels [54].

A promising field is that regarding genetic and epigenetic dysregulation following early anesthesia. For example, Cabrera et al. described a possible altered pattern that can result in the wrong functioning of the histone acetylation process and in DNA hypomethylation, thus leading, for example, to incorrect synaptic neurotransmission, an alteration of the dendritic spine density or mitochondrial damage. A key aspect of DNA hypomethylation is that those dysregulations might even be transmitted intergenerationally to anesthesia-naïve newborns [55]. At the same time, these findings open the door to pharmacological treatments of epigenetic modifications, which can be an innovative therapeutic tool.

The limitation of this paper is mainly related to the absence of a meta-analysis due to the very different outcomes evaluated. A solution to this limitation might be provided by new prospective studies, which could determine the optimal tool to measure neurodevelopmental delay. Interesting progress, in this sense, is the partnership set by the FDA with the International Anesthesia Research Society (IARS), from which was born the foundation for the Strategies for Mitigating Anesthesia-Related neuroToxicity in Tots (SmartTots) [56]. This project will allow for the central coordination of research in this field, providing a better use of the resources at disposal. Another critical aspect to be studied concerns the different types of anesthetics used. For example, a recent review by Apai et al. focused on the neurotoxicity potential of sevoflurane, which has been linked to important neurodevelopmental deficits, especially if used for longer periods of time [57]. At the same time, halothane used to be the most common anesthetic, and now it is no longer used in daily clinical practice. Recently, many studies have been conducted on novel anesthetics to be introduced into pediatric surgical interventions, such as remimazolam (an analog of midazolam), etomidate analogs and quaternary lidocaine derivatives. Some anesthetics drugs have also been reevaluated in different formulations, such as xenon and alphaxalone. However, their effects on brain development have yet to be established [58]. Certainly, this is another important issue for future research.

## 5. Conclusions

Despite the caution needed in interpreting these results, this systematic review highlights that controlled studies on mGA administered before 4 years of age agree that there is a higher risk of neurodevelopmental delay in children receiving them. Therefore, it is recommended to carefully evaluate the risks and benefits of administering several GAs in this window of potential danger, especially in those cases where a regional or local anesthesia could be an alternative. On the other hand, it is evident that further studies are needed to substantiate the correlation between mGA and anesthesia-related neurotoxicity.

## Figures and Tables

**Table 1:** Summary of study characteristics and results. CI: confidence interval.

Author	Year	No. of Multiply Exposed Children	No. of Unexposed Children	Age at Exposure	Outcome	Results	*p*-Value
Wilder	2009	100 children had 2 GAs	4764	Before 4 y.o.	Learning disability, measured with the Minnesota regression formula, issued by the Minnesota Department of Education	HR = 1.59, 95% CI: 1.06–2.37	*p* < 0.001
		44 children had at least 3 GAs				HR = 2.60, 95% CI: 1.60–4.24	
DiMaggio	2011	71 children had 2 GAs	10,146	Before 3 y.o.	Diagnosis of developmental and behavioral disorders according to ICD-9-CM codes	HR = 2.8, 95% CI: 2.5–3.1	*p* < 0.05
		23 children had at least 3 GAs				HR = 4.0, 95% CI: 3.5–4.5	*p* < 0.05
Ing	2012	52	1523	Before 3 y.o.	CELF, Raven’s CPM, MAND and CBCL tests	Statistically significant impairment of total language (HR = 2.68, 95% CI: 1.07–6.72) and receptive language (HR = 3.52, 95% CI: 1.38–9.00), while cognition and abstract reasoning abilities were impaired but did not reach statistically significance.	Statistically significant (*p* < 0.05) for total language and receptive language
Sprung	2012	64	5007	Before 2 y.o.	ADHD prevalence	Children exposed to at least 2 GA had a higher risk of developing ADHD (HR = 1.95, 95% CI: 1.03–3.71).	*p* < 0.05
Graham	2016	620	13,586	Before 4 y.o.	EDI questionnaire	Overall EDI score in mGA-exposed children was lower by 1.2 points compared to unexposed children (95% CI: −1.83–0.61). A subanalysis by age showed a statistically significant result when the age at exposure was 2–4 y.o. (overall result and the subdomains regarding common knowledge, language/cognitive and physical well-being).	*p* < 0.001
Glatz	2017	2897 children had 2 GAs	159,619	Before 4 y.o.	Academic performances, measured evaluating school grades at age 16 and IQ test at age 18	OR = 1.26, 95% CI: 0.94–1.70	Not statistically significant
		820 children had at least 3 GAs				OR = 1.95, 95% CI: 1.22–3.11	*p* < 0.05
MASK study	2018	206	411	Before 3 y.o.	FSIQ standard score and individual domains of neuropsychological assessments. Parental reports, such as the CLDQ, the CBCL and the Behavior Rating Inventory of Executive Function	Children exposed to mGA did not differ in intelligence quotient from unexposed children. Nevertheless, a statistically significant decrease in processing speed (3.51 lower) and motor abilities (5.53 lower) was noted. Moreover, multiply exposed children’s parents reported a significant increase in behavioral and reading disorders.	Statistically significant for processing speed and motor abilities scores (*p* < 0.05 and *p* < 0.001, respectively), as well as for parental reports
Tsai	2018	342	34,678	Before 3 y.o.	ADHD prevalence	HR = 1.71, 95% CI: 1.01–2.90	*p* < 0.05
AVON study	2020	212	12,111	Before 4 y.o.	Motor, cognitive, linguistic, educational, social and behavioral neurodevelopmental outcomes	Children exposed to at least 2 GA had a statistically significant higher risk of developing motor and socio-behavioral developmental outcomes, such as dynamic balance, manual dexterity and social communication scores.	*p* < 0.01
Feng	2020	2873 children had 2 GAs	22,914	Before 2 y.o.	Diagnosis of developmental delay according to ICD-9-CM codes	HR =1.476, 95% CI: 1.155–1.887	*p* < 0.05
		2703 children had at least 3 GAs				HR 2.222, 95% CI: 1.810–2.621	*p* < 0.001

## Data Availability

Not applicable.
